# The influence of information sources on intention changes to receive COVID-19 vaccination: A prospective cohort study in Japan

**DOI:** 10.1265/ehpm.22-00266

**Published:** 2023-02-02

**Authors:** Daisuke Hori, Tsukasa Takahashi, Yudai Kaneda, Akihiko Ozaki, Takahiro Tabuchi

**Affiliations:** 1 Occupational and Aerospace Psychiatry Group, Institute of Medicine, University of Tsukuba, 1-1-1 Tennodai, Tsukuba, Ibaraki 305-8575, Japan; 2 School of Medicine, Hokkaido University, Sapporo, Hokkaido 060-8638, Japan; 3 Department of Breast Surgery, Jyoban Hospital of Tokiwa Foundation, Iwaki, Fukushima 972-8322, Japan; 4 Cancer Control Center, Osaka International Cancer Institute, Osaka, Osaka 541-8567, Japan

**Keywords:** Cohort studies, Communication media, SARS-CoV-2, Japan, Vaccination hesitancy, Vaccines

## Abstract

**Background:**

Before the COVID-19 vaccine became available, many Japanese people were undecided about whether or not to receive them. Their decisions were keys to achieving herd immunity. The impact of the type of information source on the COVID-19 vaccine uptake decision-making process remains unclear. We aimed to investigate the association between information source usage on COVID-19 and subsequent vaccine uptake status among those who have yet to decide whether to receive vaccines from non-prioritized people for vaccination.

**Methods:**

Prospective cohort online self-administered surveys were conducted in February 2021 (T1), before the start of the mass vaccination program, and September–October 2021 (T2), when the vaccines were available to all citizens. The survey’s target population was registered monitors of an Internet research company. Participants who answered “I want to get vaccinated after waiting to see how it goes.” at T1 were eligible for analysis. The outcome variable was the COVID-19 vaccine uptake status in T2, and the predictors were 20 types of information sources, categorized based on people (family members, etc.), institutions (governments, etc.), or media (TV news, etc.). Adjusted odds ratio and 95% confidence intervals were estimated using logistic regression adjusted for possible confounders.

**Results:**

The 5,139 respondents, mean age and standard deviation was 42.8 ± 12.5, 55.7% female, were eligible for analysis. 85.7% completed vaccination (including reserved/intended people) in T2. In the multivariate logistic analysis, odds ratios (95% confidence interval) for vaccine uptake were 1.49 (1.18–1.89) for workplaces/schools, 1.81 (1.33–2.47) for LINE, 0.69 (0.55–0.86) for Internet news and 0.62 (0.48–0.82) for video sharing sites.

**Conclusions:**

The type of information source usage played an important role in the decision to vaccinate against COVID-19. Although caution is needed in interpreting the results, obtaining information from workplaces/schools and LINE was influential in promoting immunization.

**Supplementary information:**

The online version contains supplementary material available at https://doi.org/10.1265/ehpm.22-00266.

## Background

Vaccine hesitancy, the reluctance or refusal to vaccinate despite the availability of vaccines, is one of the ten threats to global health raised by the world health organization [1]. Vaccine hesitancy is a continuum between full acceptance, accepting some, delaying, refusing some, and outright refusal [2]. The state lies in a dynamic process, moving upstream or downstream depending on the individual’s obtained information, social situation, and perception of the disease risk. Longitudinal studies demonstrated that COVID-19 vaccination intention before the vaccines was available is a strong predictor of actual subsequent vaccination [3, 4]. In other words, most of those who wanted to be vaccinated were likely to receive vaccination after the start of the vaccination program, and vice versa. Among those who had been in the intermediate position of the continuum, some would later receive vaccination, and others would not. So, what factors split their decisions? In Japan, general vaccine trust is reported to be lower than in other countries [5]. The national vaccination program in Japan began in March 2021 for healthcare workers (HCW), and in April for people with underlying medical conditions or aged 65 years and older. Cross-sectional surveys conducted before the start of mass vaccination revealed that about one-third of the Japanese population was indecisive about getting or not getting COVID-19 vaccination once it was available [6, 7]. Therefore, their decision to receive vaccination determined the achievement of herd immunity.

Information sources might play a key role in the decision to vaccinate against COVID-19. It was suggested that exposure to information sources directly changes individual health behavior through (1) invoking cognitive and emotional responses, (2) lowering psychological obstacles to take action, and (3) recognizing the social norms related to the behavior [8]. Researchers investigated the relationships between vaccine hesitancy/uptake and information sources usage [6, 9–11]. Typical media, such as TV news or newspaper, were reported to be associated with a higher probability of willingness to be vaccinated [10, 11]. In contrast, obtaining information from the Internet, including video-sharing sites such as YouTube, exhibited a lower likelihood of receiving vaccination [6, 10, 11]. However, the main weakness of those studies lied in the fact that they assessed only vaccination intention and did not address the actual vaccine uptake status after the vaccines were available. Answering what kind of information sources affect the intention-action gap is critical to evaluate the effective risk communication strategies to promote vaccination at that time.

In the present study, a nationwide online prospective cohort survey was conducted in Japan. The target of the analysis is non-prioritized adults, namely non-HCW, healthy, and 18–64 years old. They have a low vaccine coverage rate and play a key role in COVID-19 transmission [12]. The aim of the current manuscript specifically sought to answer the following research question: What information source influenced the decision-making of non-prioritized people who had not determined whether to receive or refuse the COVID-19 vaccine before the start of mass vaccination?

## Methods

The data were derived from ongoing study called JASTIS (Japan Society and New Tobacco Internet Survey) and JACSIS (Japan COVID-19 and Society Internet Survey). The study profiles have been described elsewhere [13–15]. They are web-based, self-administered cohort survey using the same survey panel. The survey population consisted of approximately 2.2 million panelists at an Internet research firm (Rakuten Insight, Inc., Tokyo, Japan). In the surveys, possible respondents were drawn stratified by sex, age, and prefecture of residence to be as nationally representative as possible. We used two timepoints data to conducted from February 8th–25th 2021 (T1) when COVID-19 vaccines were not yet available, and September 27th to October 29th 2021 (T2) when COVID-19 vaccines became available to the entire adult population. All respondents received a nominal incentive for survey completion for each timepoint. The exclusion criteria were summarized in Additional file 1.

The following variables were derived from the T1 survey: sex, age group, employment status, marital status, educational background, annual household income, influenza vaccination in 2019 season. The following variables were derived from the T2 survey: information source and vaccine hesitancy scale.

In each survey, COVID-19 vaccine intention and uptake were evaluated with a single item question. The detail is described in Additional file 2. Participants who answered “I want to get vaccinated after waiting to see how it goes.” at T1 were eligible for analysis. According to the response at T2, “vaccinated/reserved/intended” group were coded as 1 for the following binomial logistic regression analysis. “Wait-and-see/refused” group was coded as 0.

To assess information use, the types of information sources based on people or institutions and media that individuals use to obtain information about COVID-19 as predictors. The participants were asked “did you get COVID-19 and other health-related information from each of the following sources?” for the following 20 types of information source categorized based on people, institutions, or media: (1) family, (2) friends, (3) workplaces/schools, (4) medical workers, (5) celebrities, (6) professionals, (7) websites of government and municipal offices, (8) websites of academic institutions, (9) video sharing sites (YouTube, etc.), (10) LINE, (11) Twitter, (12) Facebook, (13) Instagram, (14) Internet news, (15) newspapers, (16) magazines, (17) books, (18) TV news, (19) TV tabloid shows, and (20) radio. For each item, participants responded “yes” = 1 or “no” = 0. LINE (LINE Corp., Tokyo) is a messenger application with widespread popularity in Japan. LINE is a communication tool like iMessage or WhatsApp.

The vaccine hesitancy scale modified version for adults [16] which comprised nine items scored on a five-point Likert scale from 1 (strongly disagree) to 5 (strongly agree) was used. JASTIS/JACSIS group members translated it into Japanese. One item “I do not need vaccines for diseases that are not that common anymore” was omitted from original version because COVID-19 remained to be convergent. Seven items such as “Vaccines are important for my health.” were reverse scoring. The total scores, ranging from 9 to 45, were calculated. Higher total score indicates a higher level of vaccine hesitancy. The Cronbach α was 0.82.

To identify factors associated with COVID-19 vaccine uptake, we calculated adjusted odds ratio (AOR) and 95% confidence interval (CI) using a binomial logistic regression. The objective variable was COVID-19 vaccine uptake status. The explanatory variables were 20 types of information source, sociodemographic factors, flu vaccination in 2019 season, and vaccine hesitancy scale, which were forcedly entered. All statistical tests were two-tailed, with *p* values of <0.05 considered statistically significant. IBM SPSS Statistics for Windows, Version 28 (IBM Corp., Armonk, NY) was used.

## Results

**Fig. 1:**
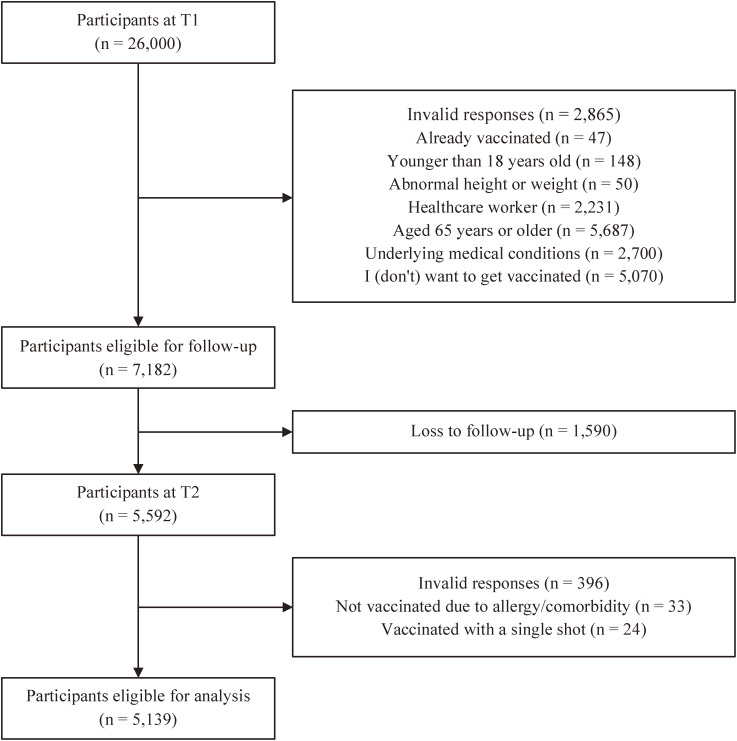
Flowchart for eligible participants for analysis. T1, February 2021. T2, September-October 2021. See Additional file 1 for exclusion criteria in detail.

Figure 1 illustrates the flowchart for selecting respondents eligible for analysis. At T1, 26,000 people participated in the survey as a whole. After excluding those who met the exclusion criteria (e.g., who had already decided to receive COVID-19 vaccine once it is available), there were 7,182 remained. At T2, 5,139 individuals (average age 42.8 ± 12.5 years at T1, 55.7% female) remained for analysis. The follow-up rate was 71.6% (= 5,139/7,182).

Supplementary table in Additional file 3 outlines the characteristics of respondents. TV news (78.8%) was the most common information source for COVID-19 related information, followed by Internet news (69.1%). Table 1 summarizes the results of the binomial logistic regression analyses. At T2, 85.7% of them were classified as vaccinated/reserved/intended group. The majority of the group already got vaccinated at least once. After adjusting for other explanatory variables, Internet news (AOR = 0.69, 95% CI = 0.55–0.86), workplaces/schools (AOR = 1.49, 95% CI = 1.18–1.89), LINE (AOR = 1.81, 95% CI = 1.33–2.47), and video sharing sites (AOR = 0.62, 95% CI = 0.48–0.82) were statistically significantly associated with vaccine uptake status. Being young, unemployed, lower educational attainment, lower household income, not receiving influenza vaccination, and higher vaccine hesitancy predicted unvaccinated status. The Nagelkerke’s R^2^ was 0.288.

**Table 1:** Prevalence of COVID-19 vaccine uptake rate by each category, and its relationship to information source usage.

**Explanatory variable**	**N**	**Vaccinated/** **reserved/** **intended, %**	**AOR^b^**	**95% CI**
TV News				
Yes	4,051	87.7	1.24	(0.96–1.61)
No	1,088	78.2	Reference	
Internet news				
Yes	3,551	86.2	0.69	(0.55–0.86)
No	1,588	84.5	Reference	
TV tabloid shows				
Yes	2,863	87.5	0.99	(0.79–1.23)
No	2,276	83.3	Reference	
Family				
Yes	2,593	87.7	0.85	(0.68–1.07)
No	2,546	83.6	Reference	
Workplaces/schools				
Yes	2,248	89.7	1.49	(1.18–1.89)
No	2,891	82.6	Reference	
Websites of government and municipal offices				
Yes	2,135	89.3	1.22	(0.99–1.51)
No	3,004	83.1	Reference	
Friends				
Yes	2,069	88.4	0.98	(0.76–1.25)
No	3,070	83.8	Reference	
Newspaper				
Yes	1,835	89.2	1.07	(0.86–1.33)
No	3,304	83.7	Reference	
Professionals				
Yes	1,832	89.5	1.20	(0.94–1.53)
No	3,307	83.6	Reference	
Medical workers				
Yes	1,033	89.9	1.17	(0.89–1.53)
No	4,106	84.6	Reference	
LINE				
Yes	881	90.7	1.81	(1.33–2.47)
No	4,258	84.6	Reference	
Twitter				
Yes	850	84.8	1.00	(0.77–1.29)
No	4,289	85.8	Reference	
Celebrities				
Yes	837	87.2	0.84	(0.63–1.12)
No	4,302	85.4	Reference	
Radio				
Yes	752	87.8	1.03	(0.77–1.37)
No	4,387	85.3	Reference	
Video sharing sites, such as YouTube				
Yes	683	81.1	0.62	(0.48–0.82)
No	4,456	86.4	Reference	
Websites of academic institutions				
Yes	419	85.4	0.71	(0.49–1.03)
No	4,720	85.7	Reference	
Magazines				
Yes	366	87.4	1.01	(0.65–1.56)
No	4,773	85.5	Reference	
Instagram				
Yes	288	84.0	0.74	(0.48–1.15)
No	4,851	85.8	Reference	
Books				
Yes	244	82.8	0.63	(0.39–1.03)
No	4,895	85.8	Reference	
Facebook				
Yes	205	87.8	1.46	(0.84–2.53)
No	4,934	85.6	Reference	
Sex^a^				
Male	2,277	84.5	Reference	
Female	2,862	86.6	1.06	(0.87–1.29)
Age group^a^				
18–34	1,419	82.2	Reference	
35–44	1,200	84.8	1.08	(0.84–1.39)
45–54	1,445	87.5	1.36	(1.05–1.76)
55–64	1,075	88.8	1.28	(0.95–1.73)
Employment status^a^				
Employed	3,587	86.7	Reference	
Unemployed	330	72.7	0.58	(0.42–0.80)
Not working (student/homemaker/retire)	1,222	86.1	0.99	(0.78–1.26)
Marital status^a^				
Single/divorced/widowed	2,790	88.9	Reference	
Married	2,349	81.8	1.19	(0.97–1.46)
Educational background^a^				
Others	2,590	87.7	Reference	
4-year college/university/graduate	2,549	83.6	1.26	(1.05–1.52)
Household income, million yen^a^				
Less than 5	1,739	81.5	Reference	
5–10	1,698	89.2	1.48	(1.17–1.85)
10 and over	535	91.0	1.60	(1.12–2.30)
I don’t know. /Prefer not to answer.	1,167	84.2	1.30	(1.04–1.63)
Flu vaccination in 2019 season^a^				
No	1,600	92.8	Reference	
Yes	3,539	82.5	2.11	(1.68–2.65)
Vaccine hesitancy scale				
Low, 9–21	1,593	97.5	3.23	(2.25–4.62)
Middle, 22–25	1,897	91.8	Reference	
High, 26–45	1,649	67.2	0.20	(0.16–0.24)

AOR, adjusted odds ratio; CI, confidence interval.

^a^The variables were assessed at T1, February 2021. The other variables were assessed at T2, September–October 2021.

^b^Logistic regression analysis were used. The objective variable was vaccine uptake status. “Vaccinated/reserved/intended” group was coded as 1. “Wait-and-see/refused” group was coded as 0.

Information sources (TV news, etc.) usage for COVID-19 and other health-related information were sorted in descending order by number of users.

## Discussion

The present study assessed the association between the various types of information use and vaccine uptake status among those who were non-prioritized and looking to see what was going on before the start of mass vaccination program. The results showed that information sources, such as “workplaces/schools” and “LINE” usage were associated with a higher probability of receiving vaccination for COVID-19. By contrast, “Internet news” and “video sharing sites” users were less likely to receive the vaccines. These information sources played a key role in providing information for deciding to receive COVID-19 vaccination or not.

As Hiraoka et al. claimed [10], workplace-based approach to promote vaccination could have worked to accelerate vaccination uptake rates. Infected and concentrated contacts persons were forced to self-isolation/quarantine, and their increased number resulted in a labor shortage. Therefore, companies worked hard to prevent COVID-19 to reduce the number of those people. Although we failed to investigate what kinds of infection control the companies made, their actions could have included providing the employees of information from public authorities. The employee’s perceived organizational support might play a role in accepting the vaccine promoting messages from their workplace [17]. It should be noted that vaccination programs in large companies have started on June 2021 (between T1 and T2). The number of first and second vaccination each reached nearly 10 million shots [18].

Most of previous studies have not addressed the specific use of LINE [6, 10]. By employing detailed categories, the present study added to the existing literature that LINE affected vaccine coverage in Japan. Under COVID-19 crisis, LINE has collaborated with the Japanese Ministry of Health, Labor and Welfare and also local governments to disseminate COVID-19-related information [19]. LINE also acts as a platform for various public service. For example, residents in some municipalities were able to reserve vaccination via LINE. Strategies to take best advantage of the capabilities of LINE could have increased COVID-19 vaccine coverage. However, not all individuals have access to LINE. In the analyzed sample, only 17.1% (= 881/5,139) obtained COVID-19 related information from the LINE.

In accordance with previous studies [6, 10], Internet news and video sharing sites usage were negatively associated with vaccine uptake. They are a great tool for disseminating information. However, they have the drawback that the information obtained is easily biased [20]. People often choose content that supports their opinions when seeking information on vaccines. Moreover, the search history will suggest the person’s preferred, similar to already accessed contents. Therefore, if a person who were indecisive about vaccination searches health information by the Internet, their choice to keep their distance to vaccines would be reinforced easily. Since it is not practical to deprive people of access to the Internet, it is important to provide information through a variety of channels other than the Internet.

It was also observed that there was a considerable range in the use of information sources among the individuals analyzed (from 4.0% of Facebook to 78.8% of TV news). Our results have implications for public health authorities to effectively communicate with the target population. The public health authorities must admit that less than half of them get information directly from the websites of government and municipal offices. When promoting immunization programs, it was important to reach out through multiple information sources which were familiar with them. Workplaces/schools and LINE could be the promising candidates for disseminating information.

The present study has some limitations. First, information source usage was only assessed at T2. Thus, cross-sectional analytical design was employed and could not draw conclusions on the direction of causality. Second, the information source usage was simply answered with yes or no. It is unclear how frequent, and what kind of information the respondents get from them. Third, the participants were the registered panelist of the Internet research company, which may have led to selection bias. They might be more familiar with Internet than the general population. Fourth, the nature of the self-administered survey may have led to recall bias and reporting bias. Fifth, reliability and validity of the Japanese version of vaccine hesitancy scale have not yet been documented. Lastly, the social context regarding the COVID-19 vaccines is fluid. Caution is needed in generalizing the current result to other periods.

## Conclusion

This study suggests that, depending on the source of health information, there is a variation in the change in deciding the intention for COVID-19 vaccination among those who were undecided about their attitudes. Those who obtained information from workplaces/schools and LINE were likely to get immunized, whereas Internet news and video sharing sites users were unlikely to. Our results emphasized the importance of recognizing and understanding information sources they used in promoting the vaccination.

## Abbreviations

AOR: Adjusted odds ratio
CI: Confidence interval
COVID-19: Coronavirus disease of 2019
HCW: Healthcare worker
JACSIS: Japan COVID-19 and Society Internet Survey
JASTIS: Japan Society and New Tobacco Internet Survey
SARS-COV-2: Severe acute respiratory syndrome coronavirus 2

## Supplementary information

**Additional file 1:** Exclusion criteria.

**Additional file 2:** Evaluation of COVID-19 vaccine intention and uptake status.

**Additional file 3:** **Supplementary table** Characteristics of the respondents at each timepoint.

## Declarations

### Availability of data and material

The data that support the findings of this study are available from the corresponding author upon reasonable request.
