# Supplementary material for: The influence of information sources on intention changes to receive COVID-19 vaccination: A prospective cohort study in Japan
Source: Environ Health Prev Med. 2023 Feb 2;28:10. doi: 10.1265/ehpm.22-00266 (PMC9922561; doi:10.1265/ehpm.22-00266)
Supplement: Supplementary file 1 — Additional file 1: Exclusion criteria. [file ehpm-28-010-s001.docx]

**Exclusion criteria**

Respondents who had provided invalid responses for three conditions were excluded at T1 and T2: 1) those who did not follow the dummy instruction, “choose the second option from the bottom of a list”, 2) those who answered they sometimes or almost every day use all of the following nine substances: alcohol, sleeping pills/antianxiety drug, medical narcotics for cancer pain, medical narcotics for all pain except cancer, non-medical narcotics, organic solvents, dangerous drugs, marijuana, and stimulant/cocaine/heroin; or 3) those who answered they currently had all of the following nine comorbidities: hypertension, diabetes mellitus, asthma, atopic dermatitis, angina pectoris, myocardial infarction, stroke/cerebral infarction/cerebral hemorrhage, cancer/malignant tumor, and chronic pain.

At T1, those who said they had already been vaccinated, those who were younger than 18 years old, those who had abnormal weight (less than 30 kg, or 150 kg and above) or abnormal height (less than 130 cm) were excluded.

Prioritized group, such as healthcare workers, those who were 65 years and older, those who were 18-64 years old with at least one pre-existing condition were also excluded. Pre-existing conditions were defined as the following fourteen conditions: hospital attendance for hypertension, diabetes mellitus, asthma, pneumonia/bronchitis, angina pectoris, myocardial infarction, stroke (including cerebral infarction and cerebral hemorrhage), chronic obstructive pulmonary disease, chronic kidney disease, chronic liver disease (excluding fatty liver and hepatitis), immune disorders and other diseases that cause immune deficiency (including steroid use), or cancer (including malignant tumor); have had sleep apnea identified by medical checkup or physician; had a BMI of 30 or over.

Respondents who answered “I want to get vaccinated.” or “I don’t want to get vaccinated.” regarding COVID-19 vaccine intention were also excluded.

At T2, those who said they would not get vaccinated because of an allergy/comorbidity and those who had received a single-shot type vaccine were excluded.
