# Supplementary material for: The influence of information sources on intention changes to receive COVID-19 vaccination: A prospective cohort study in Japan
Source: Environ Health Prev Med. 2023 Feb 2;28:10. doi: 10.1265/ehpm.22-00266 (PMC9922561; doi:10.1265/ehpm.22-00266)
Supplement: Supplementary file 2 — Additional file 2: Evaluation of COVID-19 vaccine intention and uptake status. [file ehpm-28-010-s002.docx]

**Evaluation of COVID-19 vaccine intention and uptake status.**

**---------------------------------------------------------------------------------------------------------**

At T1, February 2021, COVID-19 vaccine intention was evaluated with a single item question, “What is your opinion about COVID-19 vaccination?” The participants chose one from the three options.

In the current manuscript, the respondents who answered “1. I don’t want to get vaccinated.” or “3. I want to get vaccinated.” were excluded from the analysis.

1. I don’t want to get vaccinated.
2. I want to get vaccinated after waiting to see how it goes.
3. I want to get vaccinated.

---------------------------------------------------------------------------------------------------------

At T2, September-October 2021, COVID-19 vaccine uptake status was evaluated with a single item question, “Answer your COVID-19 vaccine uptake status.” The participants chose one from the eight options.

In the current manuscript, the respondents who answered “4. Although I would like to get vaccinated, I'm unable to do so due to allergy/comorbidity.” or “5. I have had one dose of vaccine (single-shot type).” were excluded from the analysis.

The respondents were dichotomized into the followings:

Wait-and-see/refused group, who answered 1. or 2.

Vaccinated/reserved/intended group, who answered 3., 6., 7., or 8.

1. I don’t want to get vaccinated.
2. I want to get vaccinated after waiting to see how it goes.
3. I want to get vaccinated / I have reserved my first vaccination dose
4. Although I would like to get vaccinated, I'm unable to do so due to allergy/comorbidity.
5. I have had one dose of vaccine (single-shot type).
6. Although I have had my first dose of vaccine, I did not get my second dose.
7. I have had first dose of vaccine, and am waiting for my second dose.
8. I have had two doses of vaccine.

---------------------------------------------------------------------------------------------------------
