# Supplementary material for: The influence of information sources on intention changes to receive COVID-19 vaccination: A prospective cohort study in Japan
Source: Environ Health Prev Med. 2023 Feb 2;28:10. doi: 10.1265/ehpm.22-00266 (PMC9922561; doi:10.1265/ehpm.22-00266)
Supplement: Supplementary file 3 — Additional file 3: Supplementary table Characteristics of the respondents at each timepoint. [file ehpm-28-010-s003.docx]

Supplementary table

Characteristics of the respondents at each timepoint.

|  |  | T1, February 2021 | |  | T2, September-October 2021 | |
| --- | --- | --- | --- | --- | --- | --- |
| Explanatory variable | | N | % |  | N | % |
|  |  |  |  |  |  |  |
| Prior vaccine intention | |  |  |  |  |  |
|  | Intend | 0 | 0 |  | — | — |
|  | Wait-and-see | 5,139 | 100 |  | — | — |
|  | Refuse | 0 | 0 |  | — | — |
| Vaccine uptake status | |  |  |  |  |  |
|  | Vaccinated/reserved/intended | — | — |  | 4,403 | 85.7 |
|  | Wait-and-see/refuse | — | — |  | 736 | 14.3 |
| TV News | |  |  |  |  |  |
|  | Yes | — | — |  | 4,051 | 78.8 |
|  | No | — | — |  | 1,088 | 21.2 |
| Internet news | |  |  |  |  |  |
|  | Yes | — | — |  | 3,551 | 69.1 |
|  | No | — | — |  | 1,588 | 30.9 |
| TV tabloid shows | |  |  |  |  |  |
|  | Yes | — | — |  | 2,863 | 55.7 |
|  | No | — | — |  | 2,276 | 44.3 |
| Family | |  |  |  |  |  |
|  | Yes | — | — |  | 2,593 | 50.5 |
|  | No | — | — |  | 2,546 | 49.5 |
| Workplaces/schools | |  |  |  |  |  |
|  | Yes | — | — |  | 2,248 | 43.7 |
|  | No | — | — |  | 2,891 | 56.3 |
| Websites of government and municipal offices | |  |  |  |  |  |
|  | Yes | — | — |  | 2,135 | 41.5 |
|  | No | — | — |  | 3,004 | 58.5 |
| Friends | |  |  |  |  |  |
|  | Yes | — | — |  | 2,069 | 40.3 |
|  | No | — | — |  | 3,070 | 59.7 |
| Newspaper | |  |  |  |  |  |
|  | Yes | — | — |  | 1,835 | 35.7 |
|  | No | — | — |  | 3,304 | 64.3 |
| Professionals | |  |  |  |  |  |
|  | Yes | — | — |  | 1,832 | 35.6 |
|  | No | — | — |  | 3,307 | 64.4 |
| Medical workers | |  |  |  |  |  |
|  | Yes | — | — |  | 1,033 | 20.1 |
|  | No | — | — |  | 4,106 | 79.9 |
| LINE |  |  |  |  |  |  |
|  | Yes | — | — |  | 881 | 17.1 |
|  | No | — | — |  | 4,258 | 82.9 |
| Twitter | |  |  |  |  |  |
|  | Yes | — | — |  | 850 | 16.5 |
|  | No | — | — |  | 4,289 | 83.5 |
| Celebrities | |  |  |  |  |  |
|  | Yes | — | — |  | 837 | 16.3 |
|  | No | — | — |  | 4,302 | 83.7 |
| Radio | |  |  |  |  |  |
|  | Yes | — | — |  | 752 | 14.6 |
|  | No | — | — |  | 4,387 | 85.4 |
| Video sharing site, e.g., YouTube | |  |  |  |  |  |
|  | Yes | — | — |  | 683 | 13.3 |
|  | No | — | — |  | 4,456 | 86.7 |
| Websites of academic institutions | |  |  |  |  |  |
|  | Yes | — | — |  | 419 | 8.2 |
|  | No | — | — |  | 4,720 | 91.8 |
| Magazines | |  |  |  |  |  |
|  | Yes | — | — |  | 366 | 7.1 |
|  | No | — | — |  | 4,773 | 92.9 |
| Instagram | |  |  |  |  |  |
|  | Yes | — | — |  | 288 | 5.6 |
|  | No | — | — |  | 4,851 | 94.4 |
| Books | |  |  |  |  |  |
|  | Yes | — | — |  | 244 | 4.7 |
|  | No | — | — |  | 4,895 | 95.3 |
| Facebook | |  |  |  |  |  |
|  | Yes | — | — |  | 205 | 4.0 |
|  | No | — | — |  | 4,934 | 96.0 |
| Sex |  |  |  |  |  |  |
|  | Male | 2,277 | 44.3 |  | — | — |
|  | Female | 2,862 | 55.7 |  | — | — |
| Age group | |  |  |  |  |  |
|  | 18-34 | 1,419 | 27.6 |  | — | — |
|  | 35-44 | 1,200 | 23.4 |  | — | — |
|  | 45-54 | 1,445 | 28.1 |  | — | — |
|  | 55-64 | 1,075 | 20.9 |  | — | — |
| Employment status | |  |  |  |  |  |
|  | Employed, non-HCW | 3,587 | 69.8 |  | — | — |
|  | Unemployed | 330 | 6.4 |  | — | — |
|  | Not working (student/homemaker/retire) | 1,222 | 23.8 |  | — | — |
| Marital status | |  |  |  | — | — |
|  | Single/divorced/widowed | 2,790 | 54.3 |  |  |  |
|  | Married | 2,349 | 45.7 |  | — | — |
| Educational background | |  |  |  | — | — |
|  | Others | 2,590 | 50.4 |  |  |  |
|  | 4-year college/university/graduate | 2,549 | 49.6 |  | — | — |
| Household income, million yen | |  |  |  | — | — |
|  | Less than 5 | 1,739 | 33.8 |  |  |  |
|  | 5-10 | 1,698 | 33.0 |  | — | — |
|  | 10 and over | 535 | 10.4 |  | — | — |
|  | I don't know. /Prefer not answer. | 1,167 | 22.7 |  | — | — |
| Flu vaccination in 2019 season | |  |  |  | — | — |
|  | No | 1,600 | 31.1 |  |  |  |
|  | Yes | 3,539 | 68.9 |  | — | — |
| Vaccine hesitancy scale | |  |  |  | — | — |
|  | Low, 9-21 | — | — |  | 1,593 | 31.0 |
|  | Middle, 22-25 | — | — |  | 1,897 | 36.9 |
|  | High, 26-45 | — | — |  | 1,649 | 32.1 |

Information sources (TV news, etc.) usage for COVID-19 and other health -related information were sorted in descending order by number of users.
